# Carotid Artery Perivascular Adipose Tissue Density Relates to Recanalization and Clinical Outcome After Mechanical Thrombectomy

**DOI:** 10.3389/fnagi.2021.761248

**Published:** 2021-11-24

**Authors:** Jiaolei Jin, Rui Huang, Qiuyue Chen, Boxi Ke, Taotao Tao, Rong Zhao, Xinwei He

**Affiliations:** ^1^Department of Neurology, Taizhou Central Hospital (Taizhou University Hospital), Taizhou, China; ^2^Department of Neurology, Shanghai Ninth People’s Hospital, Shanghai Jiao Tong University School of Medicine, Shanghai, China

**Keywords:** perivascular adipose tissue (PVAT), stroke, thrombectomy, carotid artery, outcome

## Abstract

**Background:** Perivascular adipose tissue (PVAT) imaging can be used in clinical practice as a surrogate marker of vascular disease. We aimed to analyze the association between the density of carotid artery PVAT and clinical features and outcomes in stroke patients treated with mechanical thrombectomy.

**Methods:** A total of 183 consecutive patients treated with mechanical thrombectomy due to anterior circulation large vessel occlusion were retrospectively included from January 2016 to May 2021. The density of carotid artery PVAT was evaluated by preoperative computed tomography angiography. Successful arterial recanalization was defined as a modified Thrombolysis in Cerebral Infarction score of 2b-3 on the final angiographic examination. Poor functional outcome was defined as a modified Rankin Scale (mRS) score > 2 at 3 months after stroke. We assessed the independent effect of carotid artery PVAT density on revascularization, functional outcome, and mortality using logistic regression models adjusted for relevant confounders.

**Results:** Patients with large artery atherosclerotic stroke have higher carotid artery PVAT density than patients with other stroke etiologies (–65.82 ± 12.96 vs. –75.77 ± 13.44, *P* < 0.001). Higher carotid artery PVAT density was associated with unsuccessful recanalization [adjusted odds ratio (AOR) (95% CI), 2.968 (1.292, 6.819), *P* = 0.010], and poor outcome [AOR (95% CI), 2.704 (1.610, 4.541), *P* < 0.001] and mortality [AOR (95% CI), 1.894 (1.040, 3.449), *P* = 0.037] at 3 months in stroke patients treated with thrombectomy.

**Conclusion:** Higher carotid artery PVAT density before mechanical thrombectomy is an indicator of worse postprocedural arterial revascularization and a worse functional outcome in acute stroke patients.

## Introduction

Acute ischemic stroke is one of the main causes of disability and mortality worldwide (Chen et al., 2020; GBD 2019 Diseases and Injuries Collaborators, 2020). Mechanical thrombectomy has been established as a standard treatment for patients with acute large vessel occlusion (Sarraj et al., 2019; Chen et al., 2020; GBD 2019 Diseases and Injuries Collaborators, 2020). The functional prognosis of some patients is still not ideal despite the successful opening of occluded vessels, suggesting that specific patient characteristics may have a significant impact on the therapeutic benefit of postoperative functional outcomes.

Perivascular adipose tissue (PVAT) is the adipose tissue depot that surrounds most blood vessels; it juxtaposes the vascular adventitia without an anatomic barrier, permitting it to directly communicate with vascular cells (Drosos et al., 2019; Kim et al., 2020). It can have both protective and deleterious effects on vessels depending on the pathophysiological environment and spatial distribution (Antonopoulos et al., 2017; Numaguchi et al., 2019). Recent studies have shown that PVAT imaging can be used in clinical practice as a surrogate marker for vascular disease (Antonopoulos et al., 2017).

The value and clinical relevance of carotid artery PVAT remain elusive and need to be clarified. A previous study showed that PVAT density is increased in the stenotic internal carotid artery (ICA) compared with the non-stenotic side in a given patient (Baradaran et al., 2018). In addition, the density of PVAT is a marker of carotid atherosclerotic plaque instability, which is a risk factor for ischemic stroke (Saba et al., 2020).

At present, there are limited data on the effect of carotid artery PVAT density on clinical prognosis after mechanical thrombectomy. Therefore, the present study aimed to analyze the association between the density of carotid artery PVAT and clinical features and outcomes in stroke patients treated with thrombectomy.

## Materials and Methods

### Study Population

We retrospectively reviewed mechanical thrombectomy databases maintained at Taizhou Central Hospital and Shanghai Ninth People’s Hospital from January 2016 to May 2021. Patients with anterior circulation large vessel occlusion (ICA, middle cerebral artery (MCA) segments M1 and M2) according to preoperative head and neck computed tomography angiography (CTA) were included. The exclusion criteria were as follows: (1) mRS score before onset of less than 2; (2) history of cerebrovascular disease with obvious sequelae; (3) poor quality or missing images.

### Mechanical Thrombectomy Therapy

Mechanical thrombectomy was generally performed based on the guideline of each era. After successful local anesthesia, femoral artery was punctured to determine the occlusion site of arteries. The microcatheter was navigated to reach the distal end of the thrombus. According to the diameter of the lesion, Solitaire™ AB embolization stents (EV3, United States) 4–6 mm in diameter and 15–30 mm long were selected as appropriate. The stent was introduced into the distal end of the occlusion and released. Then, the stent was slowly withdrawn and thrombus were removed. When necessary, the procedure was performed several times and an angiogram was performed to determine recanalization. The modified Thrombolysis in Cerebral Infarction (mTICI) score was used to assess revascularization at the end of the procedure. A mTICI score of 2b-3 was considered indicative of successful recanalization (Mohammaden et al., 2021).

### Data Collection and Outcome Assess

Demographic information, clinical features and vascular risk factors were extracted from the patients’ medical records. Additionally, regarding the treatment procedure, the occlusion site, number of passes, time from onset to revascularization, intravenous thrombolysis and periprocedural complications were registered.

The following vascular risk factors were identified: hypertension (systolic/diastolic blood pressure > 140/90 mmHg over repeated measurements or use of antihypertensive agents), diabetes mellitus (fasting blood glucose > 7.0 mmol/L, hemoglobin A1c > 6.5%, self-reported diabetes mellitus, or the use of hypoglycaemics), and hyperlipidemia (serum triglycerides > 1.7 mmol/L, low-density lipoprotein > 3.4 mmol/L, high-density lipoprotein cholesterol < 0.8 mmol/L, or the use of antihyperlipidemic agents).

Stroke etiology was determined using the Trial of ORG 10172 in Acute Stroke Treatment (TOAST) criteria: (1) large artery atherosclerosis (LAA); (2) small vessel occlusion (SVO); (3) cardioembolism (CE); and (4) other determined or undetermined etiologies (Adams et al., 1993). Neurological severity was measured with the National Institute of Health Stroke Score (NIHSS) (Adams et al., 1999), and the Alberta Stroke Programme Early CT score (ASPECTS) was determined as previously reported (Mohammaden et al., 2021). Symptomatic intracranial hemorrhage (sICH) was defined according to the European Cooperative Acute Stroke Study (ECASS) III criteria (Hacke et al., 2008).

Clinical functional outcomes were assessed at 3 months after stroke according to the score on the modified Rankin Scale (mRS), which was administered by a specialized research nurse. Poor functional outcome was defined as mRS > 2 (Aly et al., 2021).

### Imaging Acquisition and Perivascular Adipose Tissue Density Analysis

Emergency CTA was performed at our hospital using the same type of scanner with 64-slice Discovery CT750 HD (GE, United States) and the following parameters: 100 kVp, 3 mAs, section thickness 0.625 mm, interval 0.625 mm, and display field of view (DFOV) 250 × 250 mm. An angiographic phase was obtained with the administration of 1.5–2.0 mL/kg iodinated contrast (Hengrui Medicine Co., Ltd., China) injected at a flow rate of 4.0 mL/s.

**FIGURE 1:**
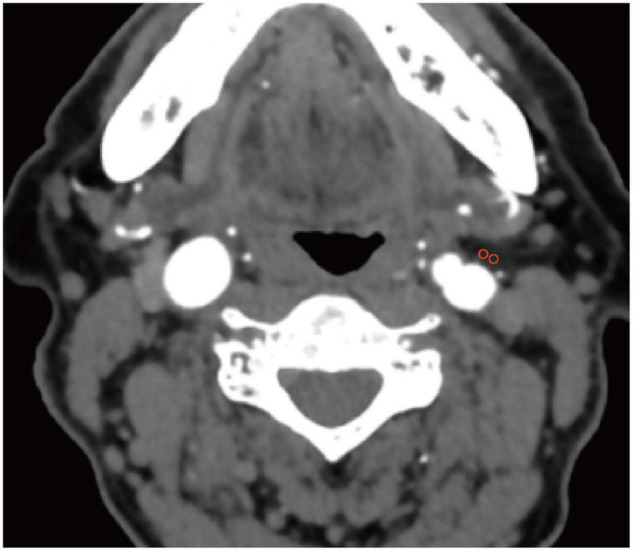
Carotid artery perivascular adipose tissue analysis. Two regions of interest (3 mm^2^ in diameter) were placed in the perivascular adipose tissue on the thrombectomy side of the origin of internal carotid artery.

An established approach using predefined image display settings (window width, 500 HU; window center, 100 HU) identified pixels corresponding to adipose tissue (Baradaran et al., 2018). Two regions of interest (ROIs) (each 3 mm^2^ in diameter) in the PVAT at the origin of the ICA on the thrombectomy side of were drawn (Figure 1) and placed at least 1 mm from the outer margin of the carotid artery wall to exclude the artery wall and surrounding soft-tissue structures. Hounsfield unit (HU) values were recorded from 3 discontinuous slices, and the average values were determined. Imaging evaluations were independently performed by two neuroradiologists who were blinded to the clinical data. The intraclass correlation coefficient (ICC) value for the two observers was 0.87 (0.76, 0.92), showing high reproducibility. The average values of the two observers were used in the final analysis.

### Statistical Analysis

Categorical data are presented as frequencies and percentages, and the chi-square test or Fisher’s exact test was used for comparison. After normality testing using the Shapiro-Wilk test, continuous variables were expressed as either the median (interquartile range) or mean ± standard deviation (SD) and compared using Student’s *t*-test, the Mann-Whitney U test or variance analysis, as appropriate. We calculated the ICC and corresponding 95% CI to evaluate the interrater reliability. Receiver operator characteristic (ROC) curve analysis was carried out to assess the accuracy of carotid artery PVAT density for predicting recanalization and outcome. Multivariate analyses were performed using logistic regression models adjusted for potential influencing factors, which were selected based on univariate analyses (*P* < 0.05). Continuous data were divided into several layers by standard deviations. All data were analyzed using SPSS 20.0 (IBM, Chicago, IL, United States). Two-sided *P* < 0.05 was considered statistically significant unless otherwise specified.

## Results

### Patient Characteristics

The study included 183 patients. The basic characteristics of the patients are shown in Table 1. A total of 119 (65.0%) patients were male, the mean age was 71.6 ± 10.0 years, the NIHSS score at baseline was 14 (10, 18), the ASPECT score at baseline was 8 (7, 9), the time from onset to recanalization was 380 min (320, 450), and the HU of the carotid artery PVAT density was –71.74 ± 14.09. 71 (38.8%) patients had an ICA occlusion, 97 (53.0%) patients had an MCA-M1 occlusion and 15 (8.2%) patients had an MCA-M2 occlusion. There was no difference in PVAT density between the three groups (*P* = 0.096).

**TABLE 1:** Characteristics of acute stroke patients treated with mechanical thrombectomy.

Variables	*n* = 183
Age, years	71.6 ( 10.0
Male, n (%)	119 (65.0%)
Risk factors, n (%)	
Hypertension	114 (62.3%)
Diabetes mellitus	47 (25.7%)
Dyslipidemia	74 (40.4%)
Coronary heart disease	56 (30.6%)
Atrial fibrillation	83 (45.4%)
Stroke history	43 (23.5%)
Hypertension med use	73 (39.9%)
Diabetes med use	32 (18.3%)
Smoking	70 (38.3%)
Drinking	26 (14.2%)
Sit of occlusion	
Internal carotid artery,n (%)	71 (38.8%)
Middle cerebral artery M1,n (%)	97 (53.0%)
Middle cerebral artery M2,n (%)	15 (8.2%)
Stroke evaluation	
NIHSS at baseline	14 (10, 18)
ASPECT score at baseline	8 (7, 9)
Successful recanalization, n (%)	166 (90.7%)
Onset to recanalization, min	380 (320, 450)
Intravenous thrombolysis, n (%)	158 (86.3%)
Number of passes	1 (1, 2)
sICH, n (%)	19 (10.4%)
TOAST subtype, n (%)	
Large-artery atherosclerosis	74 (40.4%)
Cardioembolism	87 (47.5%)
Other determined/undetermined	22 (12.1%)
HU of pericarotid fat	–71.74 ± 14.09

*NIHSS, National Institutes of Health Stroke Scale; ASPECT, Alberta Stroke Program Early CT; sICH, symptomatic intracranial hemorrhage; TOAST, Trial of Org 10172 in Acute Stroke Treatment; HU, Hounsfield Units.*

According to the TOAST etiology classification, 74 (40.4%) patients had LAA stroke, 87 (47.5%) patients had CE stroke, 22 (12.1%) patients had other determined/undetermined etiologies. Patients with LAA stroke had a higher carotid artery PVAT density than those with CE stroke (–65.82 ± 12.96 vs. –75.78 ± 14.01, *P* < 0.001). In brief, patients with LAA stroke had a higher PVAT density that other etiologies (–65.82 ± 12.96 vs. –75.77 ± 13.44, *P* < 0.001).

### Association of Carotid Artery Perivascular Adipose Tissue Density With Recanalization

**FIGURE 2:**
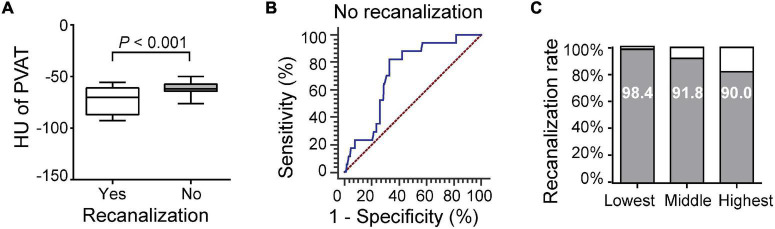
Association of carotid artery PVAT density with recanalization. **(A)** Comparison of patients with and without recanalization. **(B)** ROC analysis was performed to assess the accuracy for predicting unsuccessful recanalization. **(C)** Successful recanalization rates based on the PVAT classification. HU, Hounsfield units, PVAT, Perivascular adipose tissue.

A total of 166 (90.7%) patients achieved successful recanalization (mTICI ≥ 2b), while 17 (9.3%) did not. Of the 20 patients without recanalization, 13 had LAA stroke. Patients without recanalization had higher carotid artery PVAT density than those with recanalization (–62.14 ± 8.61 vs. –72.92 ± 14.20, *P* < 0.001, Figure 2A). ROC curve analysis was carried out to assess the prognostic accuracy of carotid artery PVAT density for unsuccessful recanalization, and the area under the curve (AUC) value was 0.722 (95% CI, 0.651–0.785; *P* < 0.001, Figure 2B). Next, we divided all patients into three categories based on the PVAT density and calculated the successful recanalization rates. The results revealed that successful recanalization was significantly associated with the carotid artery PVAT density (Figure 2C). After adjusting for differences between groups (including dyslipidemia, stroke history, ICA occlusion, intravenous thrombolysis, and number of passes, Supplementary Table 1), the difference was still statistically significant [adjusted odds ratio (AOR) (95% CI), 2.968 (1.292, 6.819), *P* = 0.010].

### Association of Carotid Artery Perivascular Adipose Tissue Density With 3-Month Functional Outcome and Mortality

**FIGURE 3:**
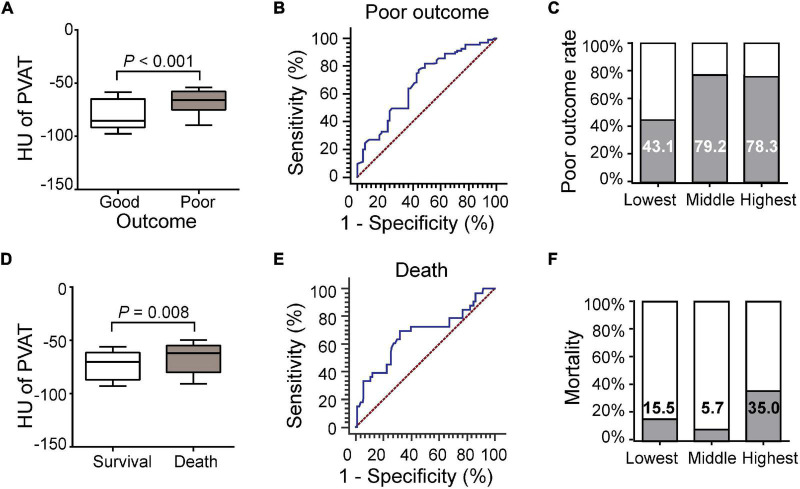
Association of carotid artery PVAT density with 3-month functional outcome and mortality. **(A)** Comparison of patients with good or poor outcomes. **(B)** ROC analysis assessing the prognostic accuracy for poor outcome. **(C)** Poor outcome rates based on the PVAT classification. **(D)** Comparison of patients with death and survival. **(E)** ROC analysis assessing the prognostic accuracy for death. **(F)** Mortality based on PVAT classification. HU, Hounsfield units, PVAT, Perivascular adipose tissue.

A total of 171 (93.4%) patients were followed up for 3 months, and 114 (66.7%) of the patients had poor outcomes. The carotid artery PVAT density in patients with poor outcomes was higher than that in patients with good outcomes (–68.37 ± 13.08 vs. –78.45 ± 14.83, *P* < 0.001, Figure 3A). ROC curve analysis revealed an AUC of 0.681 (95% CI, 0.608–0.747; *P* < 0.001, Figure 3B). Outcomes corresponded to the PVAT density (Figure 3C). After adjusting for differences between groups (including age, dyslipidemia, coronary heart disease, atrial fibrillation, smoking, ICA occlusion, and NIHSS scores, Supplementary Table 2), the difference was still statistically significant [AOR (95% CI), 2.704 (1.610, 4.541), *P* < 0.001].

Thirty-three (19.2%) of the patients died during the follow-up period. The carotid artery PVAT density of the patients who died was higher than that of the patients who survived (–65.83 ± 14.88 vs. –73.21 ± 14.05, *P* = 0.008, Figure 3D). ROC curve analysis revealed an AUC of 0.670 (95% CI, 0.594–0.740; *P* = 0.003, Figure 3E). Mortality rates did not correspond to PVAT density (Figure 3F). After adjusting for differences between groups (including stroke history, smoking, ICA occlusion, NIHSS scores, successful recanalization, intravenous thrombolysis, number of passes, and sICH, Supplementary Table 3), the difference was still statistically significant [AOR (95% CI), 1.894 (1.040, 3.449), *P* = 0.037].

## Discussion

In this study, we found that higher carotid artery PVAT density was related to the presence of LAA stroke, unsuccessful recanalization, and poor outcome and mortality at 3 months in stroke patients treated with mechanical thrombectomy.

An increasing number of studies have determined that PVAT has significant endocrine and paracrine functions that have a considerable impact on vascular structure, homeostasis and function (Drosos et al., 2019). PVAT acts as an antiatherogenic phenotype that protects against oxidation or inflammatory stimuli that trigger the onset and progression of atherosclerosis under physiological conditions (Drosos et al., 2019; Kim et al., 2020). However, PVAT contributes to the formation of atherosclerosis by increasing the release of adipocytokines and chemokines under pathological conditions, such as obesity, and diabetes (Drosos et al., 2019; Saba et al., 2020).

We found that patients with LAA stroke had higher carotid artery PVAT density than patients with other stroke etiologies. Similar to the findings of a previous clinical study, PVAT density around the stenotic ICA was higher than that around the contralateral non-stenotic ICA on the same axial slice within a given patient (Baradaran et al., 2018). The increased density of carotid artery PVAT detected with CTA imaging is closely related to histopathological markers of inflammation (Antonopoulos et al., 2017). Similarly, in humans, the PVAT around the atherosclerotic aorta has more inflammatory cells and higher pro-inflammatory gene expression than the PVAT around the healthy aorta (Verhagen et al., 2012). PVAT volume and macrophage infiltration are closely related to the size and composition of coronary atherosclerotic plaques in patients with coronary atherosclerosis (Verhagen et al., 2012). Macrophages in PVAT are more abundant in stenotic coronary arteries than in non-stenotic ones (Verhagen et al., 2014). In addition, previous basic studies have transplanted pro-inflammatory adipose tissue into normal carotid arteries, which induced atherosclerosis and increased inflammation (Ohman et al., 2011). Transplantation of thoracic PVAT from wild-type mice, which showed lower levels of inflammatory cytokines than that from *ApoE-/-* mice, nearly abrogated carotid artery plaque macrophage content without affecting plaque size (Ren et al., 2019). PVAT is located in the outermost layer of the arterial wall, which means that it participates in the pathogenesis of atherosclerosis via an outside-in mechanism, in contrast to the traditional inside-out theory of atherosclerosis (Takaoka et al., 2010). Collectively, current studies demonstrate that there is a close relationship between carotid artery PVAT and atherosclerosis, although the causal relationship has not yet been clarified.

Thus, carotid artery PVAT imaging is a useful alternative indicator for use in future carotid atherosclerosis research. In clinical practice, it is difficult to distinguish between atherosclerotic occlusion and embolic occlusion before thrombectomy. For patients with high carotid artery PVAT density, especially those without a history of heart disease, the possibility of LAA stroke should be considered. Patients with LAA stroke often have acute occlusion due to atherosclerotic stenosis. Therefore, the possible need for balloon angioplasty or stent implantation for mechanical thrombectomy should be fully considered before surgery.

We found that patients without recanalization had higher carotid artery PVAT density than those with recanalization. A recent study found that the density of PVAT around ICAs ipsilateral to the stroke or transient ischemic attack was significantly higher than that of asymptomatic ICAs (Baradaran et al., 2018). There was a positive association between perivascular fat density and vulnerable carotid atherosclerotic plaques, especially in symptomatic patients (Saba et al., 2020). The changes in PVAT differ between the initial and advanced stages of atherosclerosis, and spatial damage occurs mainly around atherosclerotic plaques (Horimatsu et al., 2018; Kim et al., 2020). In recent years, an increasing number of studies have been focused on identifying the characteristic markers related to atherosclerotic plaques. Inflammatory changes in the PVAT surrounding coronary arteries are associated with coronary artery disease and high-risk, easily ruptured vulnerable plaques (Konishi et al., 2010; Lu et al., 2016). Some studies have identified pericoronary fat density as a parameter for the evaluation of high-risk coronary plaque features (Goeller et al., 2018).

Carotid plaque composition plays a very important role in the occurrence of cerebrovascular events. Our research suggests that the increase in PVAT density around the carotid artery indicates injury to the intima and the possible existence of vulnerable plaques. The presence and number of vulnerable plaque can result in lower recanalization in patients undergoing thrombectomy. One potential mechanism may be that vulnerable plaque ruptures or lumen stenosis may block the complete removal of the thrombus. Further research is needed to elaborate this hypothesis.

We found that higher carotid artery PVAT density was associated with good 3-month outcomes and mortality after adjustment for these clinical parameters. On the one hand, it may be hypothesized that inflammatory changes in the structure and function of the upstream intracranial vessels may also affect downstream vessels, with vessels that are more distal in the brain experiencing a worse condition. On the other hand, thrombectomy causes damage to the vascular endothelium (Teng et al., 2015), and vulnerable plaques in the vascular access through which the embolectomy device passes are bound to increase the difficulty and complications of the thrombectomy operation. Such patients often require a longer procedural time and a higher number of passes and are more likely to develop *in situ* thrombosis or plaque disruption during the thrombectomy procedure. They are also more prone to secondary injury of brain tissue despite apparent complete recanalization.

Actually, many studies have focused on the value of the vascular structure for predicting the prognosis of patients after thrombectomy. For example, the distribution, pattern and degree of intracranial carotid artery calcification may be an indicator of poor outcomes, but findings are inconsistent (Kauw et al., 2021). An increasing number of studies have demonstrated that some types of carotid plaques, so-called vulnerable plaques, are likely to lead to ischemic stroke and thrombotic complications (Fabiani et al., 2020). Our findings had implications for the identification of vulnerable plaques and suggested that the features appear to extend beyond the vessel lumen of the ICA may be linked to vulnerable plaques.

Our study has limitations. First, it was a retrospective preliminary study, and our preliminary results need to be validated in a large multicenter cohort. In that case, intracranial stenosis or/and occlusion arteries can be grouped and compared more accurately. Second, the measurement of PVAT density can be affected by the change in ROI position. We chose to locate the ROI near the origin of the ICA because this location is easily, quickly and consistently identified in different individuals. Third, we measured the mean value of three discontinuous sections. It might make more sense to quantify the overall value of a certain volume.

## Conclusion

In conclusion, to the best of our knowledge, our study was the first to investigate the association between carotid artery PVAT density and clinical features and functional prognosis in stroke patients treated with thrombectomy. Our study showed that higher carotid artery PVAT density was associated with carotid atherosclerosis, worse postprocedural arterial revascularization and worse functional outcomes. Thus, evaluation of PVAT density provides opportunities for more targeted treatment and outcome evaluations.

## Data Availability Statement

The raw data supporting the conclusions of this article will be made available by the authors, without undue reservation.

## Ethics Statement

The studies involving human participants were reviewed and approved by the Ethics Committee of the Taizhou Central Hospital and Shanghai Ninth People’s Hospital. Written informed consent for participation was not required for this study in accordance with the national legislation and the institutional requirements.

## Author Contributions

RZ and XH: conception and design, interpretation of data, and revision of the manuscript. JJ, RH, and QC: writing of the manuscript and data acquisition. BK and TT: statistical analysis and interpretation of data. All authors gave final approval of the version to be published and agreed to be accountable for all aspects of the work and for any questions related to the accuracy or integrity of any part of the work.

## Conflict of Interest

The authors declare that the research was conducted in the absence of any commercial or financial relationships that could be construed as a potential conflict of interest.

## Publisher’s Note

All claims expressed in this article are solely those of the authors and do not necessarily represent those of their affiliated organizations, or those of the publisher, the editors and the reviewers. Any product that may be evaluated in this article, or claim that may be made by its manufacturer, is not guaranteed or endorsed by the publisher.
