# Supplementary material for: Carotid Artery Perivascular Adipose Tissue Density Relates to Recanalization and Clinical Outcome After Mechanical Thrombectomy
Source: Front Aging Neurosci. 2021 Nov 24;13:761248. doi: 10.3389/fnagi.2021.761248 (PMC8652413; doi:10.3389/fnagi.2021.761248)
Supplement: Supplementary file 1 [file Table_1.docx]

Supplementary Material

**Supplementary** **Table 1. Characteristics of patients between successful recanalization and no successful recanalization**

| Variables | | Successful recanalization  (n=166) | No successful recanalization  (n=17) | *P* |
| --- | --- | --- | --- | --- |
| Age, years | | 71.9 ± 10.0 | 69.1 ± 10.5 | 0.270 |
| Male, n (%) | | 110 (66.3%) | 9(52.9%) | 0.273 |
| Risk factors, n (%) | |  |  |  |
|  | Hypertension | 104 (62.7%) | 10 (58.8%) | 0.756 |
|  | Diabetes mellitus | 42 (25.3%) | 5 (29.4%) | 0.453 |
|  | Dyslipidemia | 62 (37.3%) | 12 (70.6%) | 0.008 |
|  | Coronary heart disease | 48 (28.9%) | 8 (47.1%) | 0.122 |
|  | Atrial fibrillation | 79 (47.6%) | 4 (23.5%) | 0.058 |
|  | Stroke history | 33 (19.9%) | 10 (58.8%) | < 0.001 |
|  | Hypertension med use | 66 (39.8%) | 7 (41.2%) | 0.909 |
|  | Diabetes med use | 30 (18.1%) | 2 (11.8%) | 0.514 |
|  | Smoking | 62 (37.3%) | 8 (47.1%) | 0.433 |
|  | Drinking | 22 (13.3%) | 4 (23.5%) | 0.206 |
| Stroke evaluation | |  |  |  |
|  | ICA occlusion，n(%) | 60 (36.1%) | 11 (64.7%) | 0.021 |
|  | NIHSS at baseline | 14 (10, 17) | 15 (12, 16) | 0.752 |
|  | ASPECT at baseline | 7 (6, 9) | 8 (7, 9) | 0.125 |
|  | Successful recanalisation, n (%) | / | / |  |
|  | Onset to recanalization, min | 380 (320, 450) | 370 (320, 500) | 0.857 |
|  | Intravenous thrombolysis, n (%) | 147 (88.6%) | 11 (64.7%) | 0.006 |
|  | Number of passes | 1 (1, 2) | 2 (1, 4) | 0.032 |
|  | sICH, n(%) | 16 (9.6%) | 3 (17.6%) | 0.303 |
| TOAST subtype, n (%) | |  |  |  |
|  | Large-artery atherosclerosis | 64 (38.6%) | 10 (58.8%) | 0.227 |
|  | Cardioembolism | 82 (49.4%) | 5 (29.4%) |  |
|  | Other determined/undetermined | 20 (12.0%) | 2 (11.8%) |  |

ICA, internal carotid artery, NIHSS, National Institutes of Health Stroke Scale, ASPECT, Alberta Stroke Program Early CT Score, sICH，symptomatic intracranial hemorrhage, TOAST, Trial of Org 10172 in Acute Stroke Treatment.

**Supplementary Table 2. Characteristics of patients between good and poor outcome**

| Variables | | Good outcome  (n=114) | Poor outcome  (n=57) | *P* |
| --- | --- | --- | --- | --- |
| Age, years | | 69.5 ± 9.4 | 72.9 ± 10.0 | 0.033 |
| Male, n (%) | | 35 (61.4%) | 77 (67.5%) | 0.426 |
| Risk factors, n (%) | |  |  |  |
|  | Hypertension | 34 (59.6%) | 72 (63.2%) | 0.656 |
|  | Diabetes mellitus | 10 (17.5%) | 32 (28.1%) | 0.132 |
|  | Dyslipidemia | 16 (28.1%) | 50 (43.9%) | 0.046 |
|  | Coronary heart disease | 9 (15.8%) | 42 (36.8%) | 0.005 |
|  | Atrial fibrillation | 20 (35.1%) | 59 (51.8%) | 0.039 |
|  | Stroke history | 9 (15.8%) | 32 (28.1%) | 0.076 |
|  | Hypertension med use | 21 (36.8%) | 46 (40.4%) | 0.658 |
|  | Diabetes med use | 10 (17.5%) | 18 (15.8%) | 0.770 |
|  | Smoking | 11 (19.3%) | 55 (48.2%) | < 0.001 |
|  | Drinking | 6 (10.5%) | 19 (16.7%) | 0.284 |
| Stroke evaluation | |  |  |  |
|  | ICA occlusion，n(%) | 12 (21.1%) | 55 (48.2%) | 0.001 |
|  | NIHSS at baseline | 10 (7, 14) | 15 (12, 18) | < 0.001 |
|  | ASPECT at baseline | 8 (7.5, 10) | 8 (7, 8) | 0.015 |
|  | Successful recanalisation, n (%) | / | / |  |
|  | Onset to recanalization, min | 380 (325, 450) | 380 (310, 463) | 0.870 |
|  | Intravenous thrombolysis, n (%) | 54 (94.7%) | 97 (85.1%) | 0.064 |
|  | Number of passes | 1 (1, 2) | 2 (1, 3) | 0.077 |
|  | sICH, n(%) | 1 (1.8%) | 18 (15.8%) | 0.006 |
| TOAST subtype, n (%) | |  |  |  |
|  | Large-artery atherosclerosis | 41 (36.0%) | 27 (47.3%) | 0.125 |
|  | Cardioembolism | 57 (50.0%) | 25 (43.9%) |  |
|  | Other determined/undetermined | 16 (14.0%) | 5 (8.8%) |  |

ICA, internal carotid artery, NIHSS, National Institutes of Health Stroke Scale, ASPECT, Alberta Stroke Program Early CT Score, sICH，symptomatic intracranial hemorrhage, TOAST, Trial of Org 10172 in Acute Stroke Treatment.

**Supplementary Table 3. Characteristics of patients between survival and death**

| Variables | | Survival  (n=138) | Death  (n=33) | *P* |
| --- | --- | --- | --- | --- |
| Age, years | | 72.0 ± 9.9 | 70.6 ± 9.7 | 0.456 |
| Male, n (%) | | 91 (65.9%) | 21 (63.6%) | 0.802 |
| Risk factors, n (%) | |  |  |  |
|  | Hypertension | 86 (62.3%) | 20 (60.6%) | 0.856 |
|  | Diabetes mellitus | 32 (23.2%) | 10 (30.3%) | 0.394 |
|  | Dyslipidemia | 54 (39.1%) | 12 (36.4%) | 0.769 |
|  | Coronary heart disease | 39 (28.3%) | 12 (36.4%) | 0.361 |
|  | Atrial fibrillation | 61 (44.2%) | 18 (54.5%) | 0.284 |
|  | Stroke history | 25 (18.1%) | 16 (48.5%) | < 0.001 |
|  | Hypertension med use | 51 (37.0%) | 16 (48.5%) | 0.223 |
|  | Diabetes med use | 23 (16.7%) | 5 (15.2%) | 0.833 |
|  | Smoking | 47 (34.1%) | 19 (57.6%) | 0.013 |
|  | Drinking | 17 (12.3%) | 8 (24.2%) | 0.082 |
| Stroke evaluation | |  |  |  |
|  | ICA occlusion，n(%) | 49 (35.5%) | 18 (54.5%) | 0.044 |
|  | NIHSS at baseline | 14 (10, 17) | 15 (14, 20) | < 0.001 |
|  | ASPECT at baseline | 8 (7, 9) | 8 (7, 8) | 0.138 |
|  | Successful recanalisation, n (%) | 9 (6.5%) | 8 (24.2%) | 0.002 |
|  | Onset to recanalization, min | 380 (320, 450) | 383 (325, 495) | 0.575 |
|  | Intravenous thrombolysis, n (%) | 126 (91.3%) | 25 (75.8%) | 0.013 |
|  | Number of passes | 1 (1, 2) | 2.5 (1.3, 4) | 0.001 |
|  | sICH, n(%) | 9 (6.5%) | 10 (30.3%) | < 0.001 |
| TOAST subtype, n (%) | |  |  |  |
|  | Large-artery atherosclerosis | 54 (39.1%) | 14 (42.4%) | 0.396 |
|  | Cardioembolism | 65 (47.1%) | 17 (51.6%) |  |
|  | Other determined/undetermined | 19 (13.8%) | 2 (6.0%) |  |

ICA, internal carotid artery, NIHSS, National Institutes of Health Stroke Scale, ASPECT, Alberta Stroke Program Early CT Score, sICH，symptomatic intracranial hemorrhage, TOAST, Trial of Org 10172 in Acute Stroke Treatment.
